# ­Barriers faced by medical students in seeking mental healthcare: A scoping review

**DOI:** 10.12688/mep.19115.1

**Published:** 2022-11-16

**Authors:** Maria Berliant, Nabiha Rahman, Christopher Mattice, Chirayu Bhatt, Kay-Anne Haykal

**Affiliations:** 1 The Ottawa Hospital, University of Ottawa, Ottawa, Ontario, K1H 8M5, Canada; 2 Family Medicine, University of Ottawa, Ottawa, Ontario, K1H 8M5, Canada

**Keywords:** Medical student, medical education, mental health, burnout, access, barriers, mental healthcare

## Abstract

**Background:** Medical students commonly exhibit mental health issues. Despite the availability of professionals on medical campuses, seeking help continues to be a challenge for some students. Our review aimed to identify the barriers medical students face when seeking professional mental healthcare.

**Methods:** A Medical Subject Headings (MeSH) search was created for articles using PubMed, Embase, and PsychINFO databases to identify articles specifically about medical students and their barriers to professional mental healthcare. Inclusion criteria included articles in which barriers to mental healthcare were either the primary variable or one of multiple study results. No date limits were imposed. Reviews, pilot projects, or articles that did not address barriers to mental healthcare faced by medical students or focused on veterinary or dental students were excluded. A total of 454 articles were identified and screened by title/abstract and then full text. Data were extracted from 33 articles using an independent framework. Barriers identified were compiled and reported.

**Results:** From a total of 33 articles, the most identified barriers were fear of negative effect on residency/career opportunities, fear of confidentiality breach, stigma and fear of shaming from peers, lack of perceived seriousness/normalization of symptoms, lack of time, and fear of documentation on academic record. Students also preferred to seek care outside of their institution from fear of their provider being an academic preceptor.

**Conclusions:** Many of the barriers to mental healthcare faced by medical students relate to a fear of academic and career reprisal, and fear of confidentiality breach. It appears that despite recent efforts to decrease stigma surrounding mental illness, many medical students struggle to seek appropriate support. Access to mental healthcare can be improved by increasing transparency regarding what information will be displayed on academic records, dispelling common myths about mental healthcare, and increasing awareness about resources available for medical students.

## Introduction

Physician burnout is a serious issue amongst medical professionals and has been demonstrated to start early, with 44.2% of global medical students showing signs of burnout prior to starting residency ^1– 6^. These observations have caused many medical programs to take action through the creation of wellness programs and counselling services, designed to address and curb some of the causes of burnout during medical school ^7– 10^.

Despite the alarming levels of mental health issues and burnout exhibited by medical students during their studies, stigma exists both in reporting mental health concerns and in seeking professional help ^11, 12^. This is exemplified in a study by Givens *et al*., which showed that only 22% of depressed medical students used mental health counselling services ^13^.

Many barriers to self-reporting have been hypothesized, with the most studied being the question of anonymity. The highly competitive nature of medical school compels many students to hide any perceived weakness in the eyes of their superiors or peers, and the stigma of mental illness remains pervasive in the field of medicine ^11, 14– 16^. Given the importance of this issue, there is a body of literature discussing various barriers that medical students may have when trying to seek mental healthcare. However, at the time of our literature search there is no review article that summarizes and discusses these barriers.

A scoping review of the literature was completed with the goal of compiling and showcasing the available research on barriers to medical students seeking mental healthcare. With this information, institutions can hopefully improve their wellness programs and student outreach initiatives to address these perceived obstacles and encourage medical students to seek mental healthcare.

## Methods

This scoping review is reported in line with the PRISMA-ScR guidelines ^17^.

### Stage 1- Research question

As there was no review available on the barriers to mental healthcare faced by medical students, our goal was to conduct a scoping review to showcase the available research, describe recommendations, and identify potential for future research ^18^. Thus, we developed a broad research question “What are the barriers faced by medical students to seeking professional mental healthcare?”. We defined professional mental healthcare as that which is sought from a counsellor, therapist, or physician, as opposed to self-care and peer support. Although self-care and peer support can be effective in improving mental health, they may not have the same barriers and stigma as professional care. Our sub-questions included:

1. Are there any geographic trends in the barriers faced by medical students seeking mental healthcare?
2. Are there any recommendations for addressing barriers faced by medical students seeking mental healthcare in currently available literature?

### Stage 2 - Identify relevant studies

To find studies that address our research question, we created a Medical Subject Headings (MeSH) search (MeSH, RRID:SCR_004750) and applied it to PubMed (PubMed, RRID:SCR_004846), Embase (EMBASE, RRID:SCR_001650), and PsycINFO (PsycINFO, RRID:SCR_014799) databases on June 27, 2019. We used these three databases as studies about mental health are likely to appear in databases that focus on medicine and psychology. The search terms used included variations of medical students, mental health and illness, counseling, healthcare, and therapy, as well as descriptions of barriers and help-seeking behaviors. For a full list of search terms, please refer to Table 1, Table 2 and Table 3. We did not impose publication year or country limits on our searches in order to assess for geographic variations in identified barriers. Our review included primary articles where barriers to mental healthcare were either the primary investigated variable or one of multiple study results. We excluded relevant review articles, however, did include primary studies from their references if they were not included in our original search.

**Table 1.** Search terms and results of Medline database.

Search number	Search term	Search Results
1	Mental Health/	34,303
2	exp Mental Disorders/	1,180,766
3	exp Stress, Psychological/	123,391
4	(stress* or burnout* or compassion fatigue*).ti,ab,kw.	776,766
5	(mental adj2 (health or disorder* or disease* or illness*)).ti,ab,kw.	177,080
6	mood disorder*.ti,ab,kw.	16,635
7	(depression* or depressive*).ti,ab,kw.	358,949
8	bipolar*.ti,ab,kw.	60,845
9	depress*.ti,ab,kw.	433,285
10	anxiety.ti,ab,kw.	174,796
11	psychotic disorder*.ti,ab,kw.	8,290
12	schizophrenia*.ti,ab,kw.	107,980
13	1 or 2 or 3 or 4 or 5 or 6 or 7 or 8 or 9 or 10 or 11 or 12	2,329,091
14	((barrier* or obstacle* or stigma*) adj3 (care or help* or Counseling or Psychotherap* or therapy or therapies)).ti,ab,kw.	11,647
15	((seek* or sought or pursue* or get or getting) adj3 (help* or care or service or assistan* or Counseling or Psychotherap* or therapy or therapies)).ti,ab,kw.	33,302
16	self-referral*.ti,ab,kw.	1,251
17	14 or 15 or 16	44,515
18	Students, Medical/	33,164
19	Education, Medical, Undergraduate/	22,789
20	((medical or medicine*) adj2 student*).ti,ab,kw.	39,217
21	18 or 19 or 20	62,559
22	13 and 17 and 21	142

**Table 2.** Search terms and results of PsychInfo database.

Search Number	Search Term	Search Results
1	exp mental health/	66,222
2	exp mental disorders/	855,958
3	exp stress/	115,002
4	(stress* or burnout* or compassion fatigue*).tw.	272,840
5	(mental adj2 (health or disorder* or disease* or illness*)).tw.	267,851
6	mood disorder*.tw.	17,278
7	(depression* or depressive* or bipolar* or depress* or anxiety or psychotic disorder* or schizophrenia*).tw.	534,816
8	1 or 2 or 3 or 4 or 5 or 6 or 7	1,356,613
9	((barrier* or obstacle* or stigma*) adj3 (care or help* or Counseling or Psychotherap* or therapy or therapies)).tw.	6,776
10	((seek* or sought or pursue* or get or getting) adj3 (help* or care or service or assistan* or Counseling or Psychotherap* or therapy or therapies)).tw.	26,954
11	exp help seeking behavior/	13,578
12	self-referral*.tw.	441
13	9 or 10 or 11 or 12	39,476
14	medical students/	13,366
15	medical education/	17,877
16	((medical or medicine*) adj2 student*).tw.	15,783
17	14 or 15 or 16	28,656
18	8 and 13 and 17	166
19	limit 18 to english language	154
20	exp mental health/	66,222
21	exp mental disorders/	855,958
22	exp stress/	115,002
23	(stress* or burnout* or compassion fatigue*).tw.	272,840
24	(mental adj2 (health or disorder* or disease* or illness*)).tw.	267,851
25	mood disorder*.tw.	17,278
26	(depression* or depressive* or bipolar* or depress* or anxiety or psychotic disorder* or schizophrenia*).tw.	534,816
27	20 or 21 or 22 or 23 or 24 or 25 or 26	1,356,613
28	((barrier* or obstacle* or stigma*) adj3 (care or help* or Counseling or Psychotherap* or therapy or therapies)).tw.	6,776
29	((seek* or sought or pursue* or get or getting) adj3 (help* or care or service or assistan* or Counseling or Psychotherap* or therapy or therapies)).tw.	26,954
30	exp help seeking behavior/	13,578
31	self-referral*.tw.	441
32	28 or 29 or 30 or 31	39,476
33	medical students/	13,366
34	medical education/	17,877
35	((medical or medicine*) adj2 student*).tw.	15,783
36	33 or 34 or 35	28,656
37	27 and 32 and 36	166
38	limit 37 to english language	154
39	exp mental health/	66,222
40	exp mental disorders/	855,958
41	exp stress/	115,002
42	(stress* or burnout* or compassion fatigue*).tw.	272,840
43	(mental adj2 (health or disorder* or disease* or illness*)).tw.	267,851
44	mood disorder*.tw.	17,278
45	(depression* or depressive* or bipolar* or depress* or anxiety or psychotic disorder* or schizophrenia*).tw.	534,816
46	39 or 40 or 41 or 42 or 43 or 44 or 45	1,356,613
47	((barrier* or obstacle* or stigma*) adj3 (care or help* or Counseling or Psychotherap* or therapy or therapies)).tw.	6,776
48	((seek* or sought or pursue* or get or getting) adj3 (help* or care or service or assistan* or Counseling or Psychotherap* or therapy or therapies)).tw.	26,954
49	exp help seeking behavior/	13,578
50	self-referral*.tw.	441
51	47 or 48 or 49 or 50	39,476
52	medical students/	13,366
53	medical education/	17,877
54	((medical or medicine*) adj2 student*).tw.	15,783
55	52 or 53 or 54	28,656
56	46 and 51 and 55	166
57	limit 56 to english language	154

**Table 3.** Search terms and results of Embase database.

Search Number	Search Term	Search Results
1	Mental Health/	34,303
2	exp Mental Disorders/	1,180,766
3	exp Stress, Psychological/	123,391
4	(stress* or burnout* or compassion fatigue*).ti,ab,kw.	776,766
5	(mental adj2 (health or disorder* or disease* or illness*)).ti,ab,kw.	177,080
6	mood disorder*.ti,ab,kw.	16,635
7	(depression* or depressive*).ti,ab,kw.	358,949
8	bipolar*.ti,ab,kw.	60,845
9	depress*.ti,ab,kw.	433,285
10	anxiety.ti,ab,kw.	174,796
11	psychotic disorder*.ti,ab,kw.	8,290
12	schizophrenia*.ti,ab,kw.	107,980
13	1 or 2 or 3 or 4 or 5 or 6 or 7 or 8 or 9 or 10 or 11 or 12	2,329,091
14	((barrier* or obstacle* or stigma*) adj3 (care or help* or Counseling or Psychotherap* or therapy or therapies)).ti,ab,kw.	11,647
15	((seek* or sought or pursue* or get or getting) adj3 (help* or care or service or assistan* or Counseling or Psychotherap* or therapy or therapies)).ti,ab,kw.	33,302
16	self-referral*.ti,ab,kw.	1,251
17	14 or 15 or 16	44,515
18	Students, Medical/	33,164
19	Education, Medical, Undergraduate/	22,789
20	((medical or medicine*) adj2 student*).ti,ab,kw.	39,217
21	18 or 19 or 20	62,559
22	13 and 17 and 21	142
23	exp mental health/	34,303
24	exp mental disease/	0
25	exp mental stress/	0
26	(stress* or burnout* or compassion fatigue*).ti,ab,kw.	776,766
27	(mental adj2 (health or disorder* or disease* or illness*)).ti,ab,kw	177,080
28	mood disorder*.ti,ab,kw	16,635
29	(depression* or depressive*).ti,ab,kw.	358,949
30	bipolar*.ti,ab,kw.	60,845
31	depress*.ti,ab,kw.	433,285
32	anxiety.ti,ab,kw.	174,796
33	psychotic disorder*.ti,ab,kw.	8,290
34	schizophrenia*.ti,ab,kw.	107,980
35	23 or 24 or 25 or 26 or 27 or 28 or 29 or 30 or 31 or 32 or 33 or 34	1,488,521
36	((barrier* or obstacle* or stigma*) adj3 (care or help* or Counseling or Psychotherap* or therapy or therapies)).ti,ab,kw.	11,647
37	help seeking behaviour/	588
38	((seek* or sought or pursue* or get or getting) adj3 (help* or care or service or assistan* or Counseling or Psychotherap* or therapy or therapies)).ti,ab,kw.	33,302
39	self-referral*.ti,ab,kw.	1,251
40	36 or 37 or 38 or 39	44,628
41	medical student/	31,164
42	medical education/	55,021
43	((medical or medicine*) adj2 student*).ti,ab,kw.	39,217
44	41 or 42 or 43	98,165
45	35 and 40 and 44	131
46	limit 45 to english language	129
47	limit 46 to embase [Limit no valid in Ovid MEDLINE(R), Ovid MEDLINE(R) Daily, Ovid MEDLINE(R) In-Progress, Ovid MEDLINE(R) Publisher; records were retained]	129
48	Mental Health/	34,303
49	exp Mental Disorders/	1,180,766
50	exp Stress, Psychological/	123,391
51	(stress or burnout or compassion fatigue*).ti,ab,kw.	776,766
52	(mental adj2 (health or disorder* or disease* or illness*)).ti,ab,kw	177,080
53	mood disorder*.ti,ab,kw	16,635
54	(depression* or depressive*).ti,ab,kw.	358,949
55	bipolar*.ti,ab,kw.	60,845
56	depress*.ti,ab,kw.	433,285
57	anxiety.ti,ab,kw.	174,796
58	psychotic disorder*.ti,ab,kw.	8,290
59	schizophrenia*.ti,ab,kw.	107,980
60	48 or 49 or 50 or 51 or 52 or 53 or 54 or 55 or 56 or 57 or 58 or 59	2,329,091
61	((barrier* or obstacle* or stigma*) adj3 (care or help* or Counseling or Psychotherap* or therapy or therapies)).ti,ab,kw.	11,647
62	((seek* or sought or pursue* or get or getting) adj3 (help* or care or service or assistan* or Counseling or Psychotherap* or therapy or therapies)).ti,ab,kw.	33,302
63	self-referral*.ti,ab,kw.	1,251
64	61 or 62 or 63	44,515
65	Students, Medical/	31,164
66	Education, Medical, Undergraduate/	22,789
67	((medical or medicine*) adj2 student*).ti,ab,kw.	39,217
68	65 or 66 or 67	62,559
69	60 and 64 and 68	142

### Stage 3 - Literature selection

**Figure 1.**
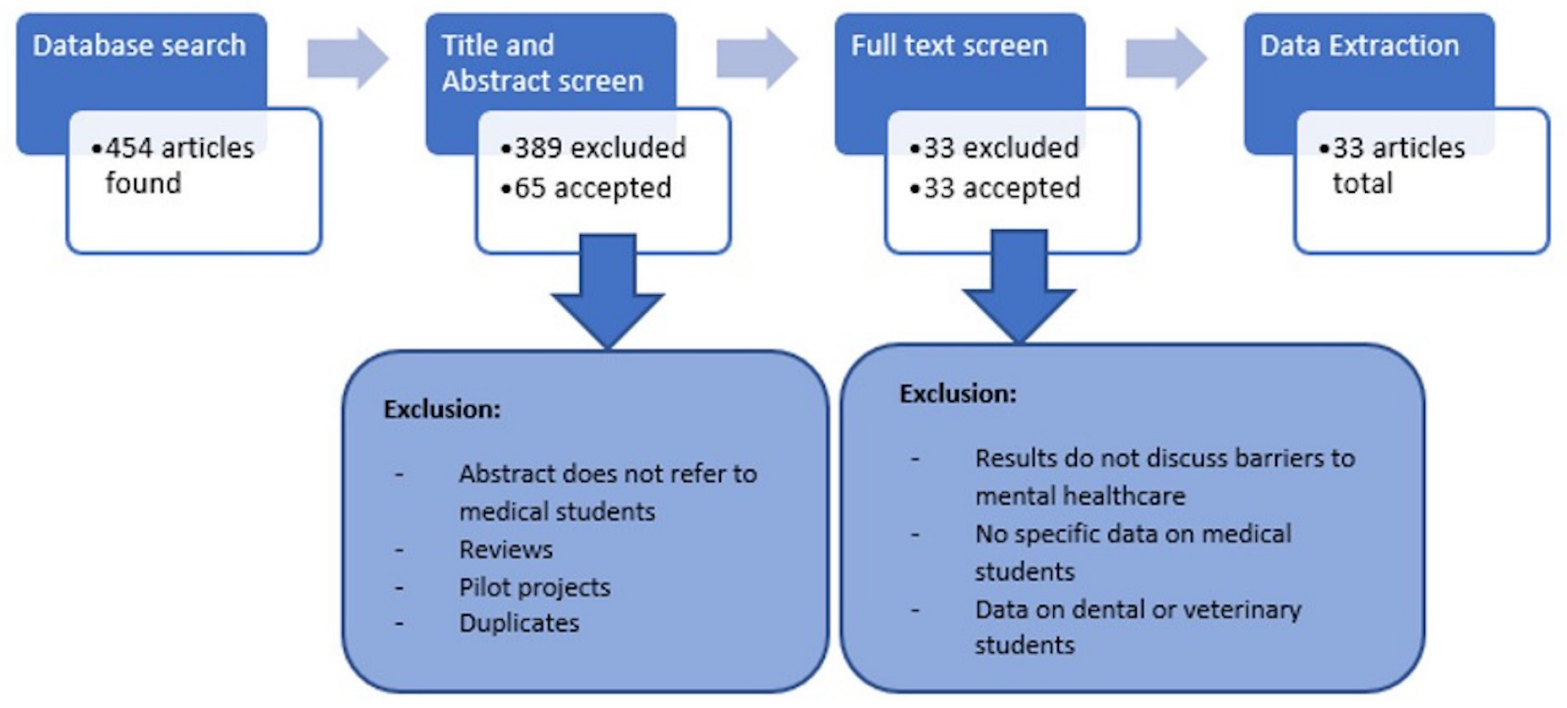
Article screening methodology.

Our literature search yielded 438 articles, and an additional 16 articles were extracted from literature reviews that were not yielded by the initial search. The resultant total of 454 articles were reviewed for title and abstract screening. Through title and abstract screening, we excluded 389 articles that were either duplicates, pilot projects, reviews, did not address barriers to mental healthcare faced by medical students, or focused on veterinary or dental students. A full-text screening performed on the remaining 65 articles excluded studies focusing on non-professional or self-care. A total of 33 articles passed the screening process and were included in our final review [ Figure 1].

### Stage 4- Charting data

Data extraction was manually performed and documented using Microsoft Excel (Microsoft Excel, RRID:SCR_016137). Our data of interest were the barriers medical students faced in seeking mental healthcare noted in each article. Exact wording from each article was used when describing barriers. Data were extracted by all authors with oversight by lead author M.B.

### Stage 5- Summarizing and reporting

Data extracted from studies were organized to answer the research question and sub-questions.

We listed barriers from all articles and compiled them within two broader categories: systemic barriers and individual barriers. Systemic barriers included “policies, procedures, or practices that unfairly discriminate and can prevent individuals from participating fully in a situation” ^19^. Individual barriers were not directly linked to institutional practices and were dictated by a person’s life experiences, emotions, and prior knowledge. Papers were assigned to barrier categories based on which barriers were identified in the study results. Studies were also categorized by geographic location, including Asia, South America, Australia, Europe, and North America. Article suggestions for addressing the barriers to medical student mental healthcare were recorded and combined to formulate an overall recommendation most applicable in North America, as this is the context of our institution.

## Results

**Figure 2.**
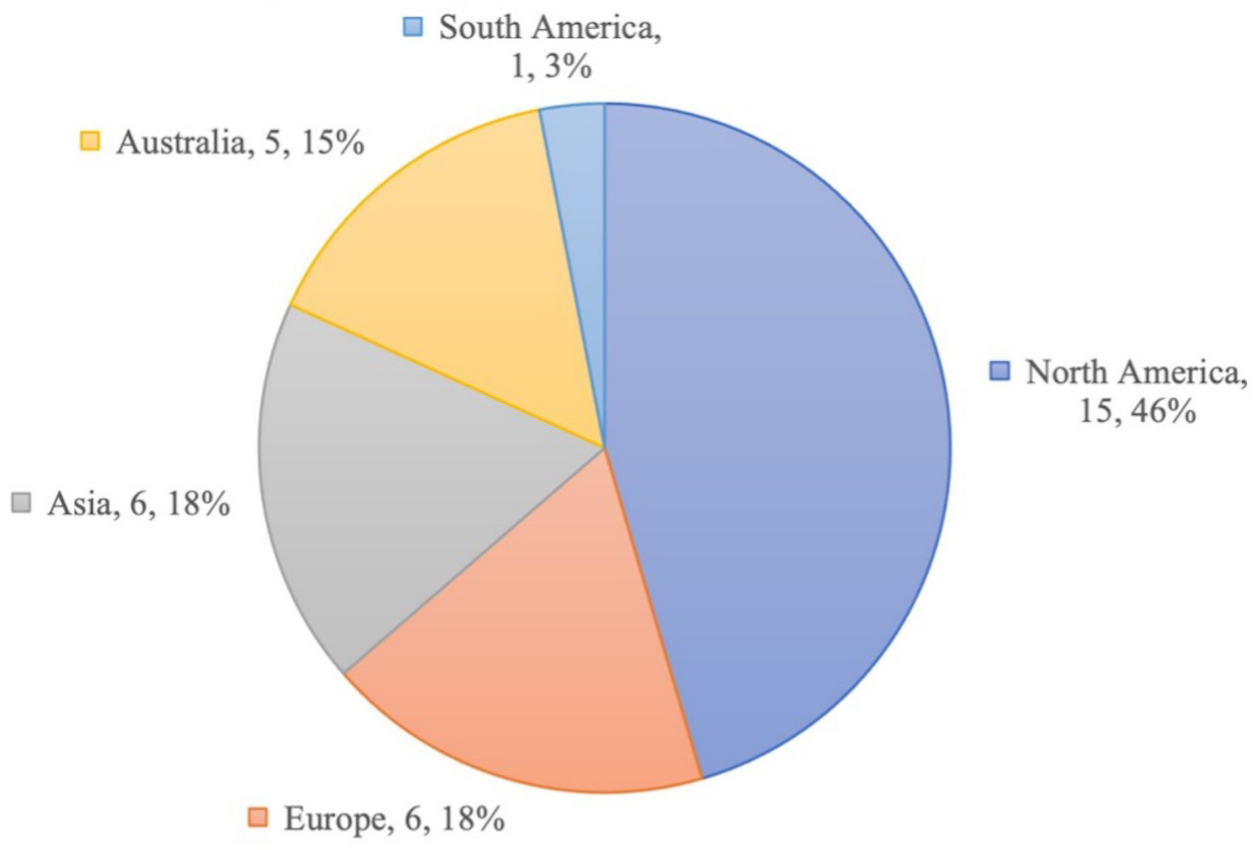
Distribution of geographical populations studied within accepted articles.

A total of 454 articles were found through our search and 33 articles were included in the data extraction portion of our study ^13, 20– 51^. Geographically, we accepted papers from all regions [ Figure 2]; most articles were studies conducted in North America (n=15, 46% total), with other regions including Europe (n=6, 18%), Asia (n=6, 18%), Australia (n=5, 15%), and South America (n=1, 3%). Barriers to seeking mental healthcare that were identified in the studies were divided into one of two categories: systemic barriers or individual barriers to mental healthcare.

### Systemic barriers

A total of nine systemic barrier types were identified and are outlined in Table 4.

**Table 4.** Systemic barriers to mental healthcare seeking by medical students.

Systemic Barrier	Total number of articles mentioning barrier	Articles from North America	Articles from Europe	Articles from Asia	Articles from Australia	Articles from South America
Affiliation of treating practitioner with university	3	2	1	0	0	0
Involvement of practitioner in medical training	5	2	1	0	2	0
Access issues	2	1	1	0	0	0
Cost	5	1	1	1	2	0
Limitation in number of sessions offered	1	1	0	0	0	0
Mandatory reporting laws	1	1	0	0	0	0
Lack of education on resources	2	0	1	0	1	0
Lack of available resources	1	0		1	0	0
Cultural stigma	2	0	1	0	1	0

The most commonly reported systemic barriers were cost (five articles) and involvement of treating practitioner with medical education (five articles). Amongst those that reported cost, one was from North America, two from Australia, one from Asia, and one from Europe. Fear of being treated by a future preceptor was reported in two articles from North America, two from Australia, and one from Europe. The systemic barriers that were reported least commonly were lack of available resources (one article), mandatory reporting laws (one article), and limitation in number of sessions offered (one article)

### Individual barriers

All 33 articles included in our review mentioned at least one individual barrier to mental healthcare faced by medical students. There were 21 individual barriers identified, outlined in Table 5.

**Table 5.** Individual barriers for seeking mental healthcare by medical students.

Individual Barrier	Total number of articles mentioning barrier	Articles from North America	Articles from Europe	Articles from Asia	Articles from Australia	Articles from South America
Personal stigma against seeking care	9	4	3	2	0	0
Fear of non-confidentiality	9	5	2	1	1	0
Fear of mental healthcare being noted on academic record	6	2	2	1	0	0
Fears of decreased opportunities for residency and career	10	5	3	0	1	0
Fear of discrimination/judgement	8	3	2	2	1	0
Lack of time to seek care	5	3	0	1	1	0
Concerns about effectiveness/ appropriateness of treatment	6	1	0	3	1	1
Felt issue may self-resolve/is not severe enough to seek care	5	3	1	1	0	0
Normalization of symptoms	4	1	1	0	1	0
Female gender	1	0	0	1	0	0
Male gender	3	2	0	0	0	1
Previous experience with mental illness in close contacts	1	0	0	1	0	0
Lack of experience with mental illness in close contacts	1	1	0	0	0	0
Lack of knowledge of resources	3	0	2	1	0	0
Preference for mental support from family, friends, peers	3	0	2	0	1	0
Competition with peers	3	0	2	1	0	0
Self-diagnosis	2	0	0	0	2	0
Diagnosed mental illness or high severity of symptoms	2	0	1	0	1	0
Fear of unwanted intervention	1	0	0	1	0	0
Fear of treatment side effects	1	0	0	1	0	0
Lack of positive mentorship	1	0	0	0	0	1

The most reported individual barrier was “Fears of decreased opportunities for residency and career”, which was cited in 10 out of 33 papers. Other commonly noted barriers were personal stigma against seeking care, fear of non-confidentiality, and fear of discrimination (general population and peers). Globally, there were differing trends in barriers, papers from North America and Europe most commonly noted a fear of decreased opportunities for residency and career, while papers from Asia most commonly noted concerns about effectiveness/appropriateness of treatment, and personal stigma against seeking care.

## Discussion

The present review aims to identify the barriers that medical students face when seeking professional mental healthcare. Our study found that medical students face several systemic and individual barriers to mental healthcare. When assessing systemic barriers, cost and affiliation of mental health practitioners with the university or involvement of practitioner in medical training were commonly noted. Prevalent individual barriers included stigma, fear of non-confidentiality, fear of impact on academic record, fear of decreased career opportunities and discrimination. We provide several recommendations on how to mitigate these issues and improve medical students' access to the mental health support they need.

### Systemic barriers

Our study found that cost was one of the most prevalent systemic barriers to seeking mental healthcare in medical students. With high tuition fees in addition to extra-curricular and personal expenses, medical students may defer mental health services due to economic reasons alone. Although cost seemed to be a common concern for North American, European and Australian medical students, it is unlikely that this is applicable to a Canadian context, as most medical schools provide mental healthcare to their students through free university counselling services. Nevertheless, Canadian students who seek counselling elsewhere may require third party financial coverage. In addition, it is uncertain whether other factors such as the students’ socioeconomic status and financial independence play a role in the likelihood of medical students seeking mental health support.

Other prevalent systemic barriers include conflict of interest with mental healthcare providers or the practitioner’s affiliation with the university. The power imbalances amongst medical students and faculty members may have prevented students from sharing their concerns with these individuals due to fear of non-confidentiality. Students were perhaps afraid of their preceptors and/or mental health practitioners revealing the students’ mental health status to future residency programs or concerned about making a negative impression.

In some cases, students may have individualized needs that the university does not specialize in, as described by the individual barrier of “fear of treatment not being appropriate” and the systemic barrier of “access issues”. For instance, an indigenous student undergoing mental health problems may benefit from a counselor who specializes in indigenous health, which may not be offered by the university. Students from certain sociocultural groups may feel underrepresented and may not be able to confide in counsellors who do not understand the cultural background or the experience dealing with these specific issues.

While a university affiliated mental healthcare service can be a very positive thing and can significantly improve access to mental healthcare for students, we must be conscious of the barriers to care that it inherently introduces. The solution is not to transfer university affiliated mental health resources to external ones, but to acknowledge the barriers and fears they may introduce for students and work to address and alleviate those concerns.

### Individual barriers

Our scoping review identified multiple individual barriers. The most commonly reported barrier was fears of decreased opportunities for residency and career with nearly 30% of articles mentioning this barrier. This fear is related to other similar individual barriers such as those of non-confidentiality and self-stigmatization, which were the second most noted barriers in our review.

Despite our best efforts, mental illness remains a heavily stigmatized topic. Medical school is challenging with high expectations and many students grapple with imposter syndrome ^52^. Experiencing a mental illness along with imposter syndrome may further heighten feelings of inadequacy and isolation, thus perpetuating self-stigmatization.

Fear of non-confidentiality also proved to be a significant concern for medical students struggling with mental illness. While confidentiality in counselling is dictated by privacy laws, the college of psychotherapists, and the Committee on Accreditation of Canadian Medical Schools (CACMS), students may still be concerned as to whether confidentiality can be maintained without knowledge of these policies. Fear of non-confidentiality goes hand in hand with the fear of reduced residency opportunities, as students may be concerned that their mental health will be reported either to licensing bodies or through the application process.

Medical student barriers to care are heavily associated with their career anxieties. For many students, residency applications and mandatory health reporting are not well understood. For students struggling with their mental health earlier in their education, it is not unreasonable to believe they may avoid seeking mental healthcare out of the fear of the unknown.

Overall, our recommendations for improved care are as follows:

### Recommendations

1. *Counselling services led by non-physicians*
  Affiliation of treating practitioners with the student’s university and fear of being treated by future preceptors and supervisors were common barriers to seeking mental health services by medical students. Hosting counselling sessions led by non-physicians may relieve the concern many students have about their current or future preceptors being within their circle of care.
2. *Transparency in residency application process*
  Medical students are often concerned that their mental health status will be disclosed and impact their future residency application. Adopting a transparent residency application process, in which students can have access to all of the information that will be disclosed on their academic record, can give students a sense of control over their application. This may mitigate the hesitancy some students may experience when sharing their personal concerns with their university’s counselling team.
3. *Encourage faculty and residents to build non-judgmental and accommodating work environment*
  Encouraging a supportive environment that facilitates academic accommodations and accepts these requests in a non-judgmental manner can relieve the competition and perceived judgement faced by students.
4. *Ensure strict confidentiality*
  Fear of non-confidentiality is one of the most prevalent barriers faced by medical students when seeking mental healthcare. It is essential to outline a strict and clear policy for maintaining confidentiality. Moreover, it is recommended to outline this policy at the start of every counselling session and to discuss concerns regarding confidentiality as they arise. In addition, medical students’ mental health concerns should remain within a pre-defined circle of care and should not be extended beyond this team.
5. *Provide non-university affiliated mental health resources*
  Despite the above measures, some students may prefer to seek support beyond their medical school due to a variety of reasons. For instance, students may have pre-existing therapeutic alliance with a non-university affiliated counsellor, or a non-university affiliated counsellor may be more accessible and/or may specialize in the specific type of support the student is seeking. Hence, it is essential to make alternate options available to students to best fit their needs.
6. *Normalize seeking mental healthcare to reduce stigma*
  In order to reduce stigma, mental healthcare should be made available to medical students as soon as they enter medical school. Moreover, conversations regarding stress-management strategies and addressing stigma towards mental health should be introduced early within the medical school orientation period.

### Limitations

There are limitations to our study and recommendations. Regarding the design, all studies included in the scoping review were found through internet searches. It is possible that some studies, especially those published in earlier years may not have a corresponding online version.

We also chose to avoid pilot projects, opinion pieces, reviews, and textbook publications; instead focusing only on primary literature. While this decision was made to improve the validity of our data, other forms of literature may have yielded additional barriers or recommendations for change.

Additionally, we limited our search to papers published in English only, but did not use geographical barriers; thus, it is possible that papers published in a different language, from a non-Western institution could have been missed in our search.

Finally, the recommendations made in our research paper are applicable primarily to a Western setting. While mental health challenges are pervasive for medical students around the world, other regions may have heightened stigma or increased healthcare service limitations. Thus, these recommendations must be implemented with appropriate cultural competence.

## Conclusions

In conclusion, while medical students often struggle with their mental health, they face significant barriers to seeking and receiving appropriate mental healthcare. Through our scoping review, we identified multiple systemic and individual barriers, with the most common systemic barriers being affiliation of counsellor with medical school, cost, and access; and the most common individual barriers being stigma, fear of non-confidentiality, fear of impact on academic record, and fear of decreased career opportunities and discrimination. Our overall recommendations included improving the transparency in the residency application process early on in medical education, ensuring strict confidentiality and student education of confidentiality laws, providing access to non-university affiliated counselling services, and working to create a positive environment without mental health stigma. We hope our review may be useful for improving medical school counseling services. Future research may focus on describing cultural differences in mental health seeking behaviors, as well as determining the success of implementing recommendations proposed in our review.

## Data Availability

All data underlying the results are available as part of the article and no additional source data are required. Figshare: PRISMA-ScR checklist for ‘Barriers faced by medical students in seeking mental healthcare: A scoping review’. https://doi.org/10.6084/m9.figshare.21508071 ^17^. Data are available under the terms of the Creative Commons Zero "No rights reserved" data waiver (CC0 1.0 Public domain dedication).
