# Supplementary material for: ­Barriers faced by medical students in seeking mental healthcare: A scoping review
Source: MedEdPublish (2016). 2022 Nov 16;12:70. [Version 1] doi: 10.12688/mep.19115.1 (PMC10331850; doi:10.12688/mep.19115.1)
Supplement: Peer review history

# Peer review: Reviewer response for version 1

**DOI:** 10.21956/mep.20473.r33774

**Published:** 2023-07-05

**Authors:** Feriha Fatima Khidri

**Affiliations:** 1 Liaquat University of Medical & Health Sciences, Jamshoro, Pakistan

Overall, I am satisfied with the writing and objectives of the article. However, I would like to suggest that authors should expand result section and in writing discussion compare the results by giving references. 

1. It is very important to mention that what was the prevalence of burn out in different studies and what kind of mental illness students suffered for e.g. anxiety, depression.
2. What were the challenges faced by students in context of mental illness?
3. Among medical students, self prescription is common especially in case of mental illness. Authors should discuss it with recent reference given below ^1^.
4. In discussion section there is just one reference. Provide discussion by comparing study results and identify differences in results among different regions or different barriers found in different region.

Are the rationale for, and objectives of, the Systematic Review clearly stated?

Yes

Is the statistical analysis and its interpretation appropriate?

Yes

Are sufficient details of the methods and analysis provided to allow replication by others?

Yes

Are the conclusions drawn adequately supported by the results presented in the review?

Yes

Reviewer Expertise:

Medical Education, Mental Health

I confirm that I have read this submission and believe that I have an appropriate level of expertise to confirm that it is of an acceptable scientific standard.

## References

[1] : Physical Activity, Dietary Habits and Factors Associated with Depression Among Medical Students of Sindh, Pakistan, During the COVID-19 Pandemic. *Psychol Res Behav Manag* .2022;15: 10.2147/PRBM.S364540 1311-1323 10.2147/PRBM.S364540 35642191PMC9148611

# Peer review: Reviewer response for version 1

**DOI:** 10.21956/mep.20473.r32930

**Published:** 2023-02-23

**Authors:** Anuradha Mishra

**Affiliations:** 1 Department of Ophthalmology, Dalhousie University, Halifax, NS, Canada

There was a very clear organization and methodology. The search terms appeared to be appropriate but I was surprised that psychiatric or psychiatry was not used as a search term.

 Given that this is pre-covid data there may be value to acknowledge this in the discussion portion. We now are living with COVID-19. How can we integrate and finding meaning with these results in a post-pandemic and increasingly virtual world?

Are the rationale for, and objectives of, the Systematic Review clearly stated?

Yes

Is the statistical analysis and its interpretation appropriate?

Not applicable

Are sufficient details of the methods and analysis provided to allow replication by others?

Yes

Are the conclusions drawn adequately supported by the results presented in the review?

Yes

Reviewer Expertise:

mixed methodology; medical education; communications skills

I confirm that I have read this submission and believe that I have an appropriate level of expertise to confirm that it is of an acceptable scientific standard.
